# Food Safety During and After the Era of COVID-19 Pandemic

**DOI:** 10.3389/fmicb.2020.01854

**Published:** 2020-08-04

**Authors:** Amin N. Olaimat, Hafiz M. Shahbaz, Nayab Fatima, Sadia Munir, Richard A. Holley

**Affiliations:** ^1^Department of Clinical Nutrition and Dietetics, Faculty of Applied Medical Sciences, The Hashemite University, Zarqa, Jordan; ^2^Department of Food Science and Human Nutrition, University of Veterinary and Animal Sciences, Lahore, Pakistan; ^3^College of Food Science and Technology, Huazhong Agricultural University, Wuhan, China; ^4^Department of Food and Human Nutritional Sciences, University of Manitoba, Winnipeg, MB, Canada

**Keywords:** coronavirus, COVID-19, SARS-CoV-2, food safety, food package, sanitation, transmission, active packaging

## Abstract

The coronavirus disease 2019 (COVID-19) is a clinical syndrome caused by severe acute respiratory syndrome corona virus-2 (SARS-CoV-2). COVID-19 was declared a pandemic by the World Health Organization (WHO) on March 11, 2020 due to its rapid and extensive spread among many countries through its very contagious nature and its high mortality among the elderly and infirm. Recently, data on the survival of SARS-CoV-2 on contact surfaces has been reported, but there is none on the survival of COVID-19 on food surfaces and packages. The potential survival and transmission of SARS-CoV-2 on/via food and packages are discussed based on data available for other respiratory viruses such as SARS-CoV and MERS-CoV. However, studies are needed to explore its transmission via food and survival on food packaging materials. The implementation of food safety management systems such as Hazard Analysis and Critical Control Points (HACCP), and Good Manufacturing Practices (GMP) are important to reduce the risk of COVID-19 infection. Cleaning, sanitation, good hygienic practices, and active packaging are also needed from farm to fork.

## Introduction

In December 2019, SARS-CoV-2 was initially detected in patients who suffered from unusual viral pneumonia in Wuhan, Hubei, China (Kaul, 2020; Naserghandi et al., 2020; Petrosillo et al., 2020). The virus was first named 2019 novel coronavirus (2019-nCoV) by the WHO and later, when it was found that 86.9% of the novel virus genome was similar to the SARS-CoV genome, the virus was renamed SARS-CoV-2 (Chang et al., 2020; The Lancet Infectious Diseases, 2020). COVID-19 is the clinical syndrome caused by SARS-CoV-2 infection which is characterized by a respiratory disease with symptoms ranging from mild influenza (flu-like) to severe pneumonia and acute respiratory distress syndrome (Petrosillo et al., 2020). The clinical manifestations of COVID-19 are non-specific and variable among patients, and between countries. Generally, COVID-19 symptoms involve fever, sore throat, runny or stuffy nose, dry cough, headache, myalgia or fatigue, sputum production, dyspnea, chest pain or pressure, joint pain, chills, loss of taste or smell, and a rash on the skin or discoloration of toes or fingers. Abdominal pain, dizziness, diarrhea, nausea, and vomiting are less common symptoms (Kaul, 2020; Naserghandi et al., 2020; Petrosillo et al., 2020). Borges do Nascimento et al. (2020) found that COVID-19-related symptoms among 59,254 patients in 61 studies were: fever (82%), cough (61%), muscle aches and/or fatigue (36%), dyspnea (26%), headache (12%), sore throat (10%), and gastrointestinal symptoms (9%). On average, the incubation period takes 5–6 days for a patient to show the symptoms after infection, however, it may reach up to 14 days (World Health Organization [WHO], 2020).

COVID-19 infection is highly contagious among the population and now almost all countries have reported cases and deaths. On March 11, 2020, COVID-19 was characterized as a pandemic by the WHO. As of early July, over 12 million confirmed cases and 550,000 deaths of COVID-19 have been reported worldwide (World Health Organization [WHO], 2020).

## COVID-19 Transmission

At the beginning of the pandemic, two-third or 27 out of 41 reported cases had previously visited the Huanan seafood wholesale market where live animals were sold close to seafood and meat products, suggesting that the virus was transmitted from animals to humans (Guan et al., 2020; Harapan et al., 2020; Naserghandi et al., 2020). The genome of SARS-CoV-2 is closely related to the genomes of the SARS-CoV that caused the SARS epidemic during 2003 and the SARS-related-CoVs (SARSr-CoVs) that was isolated from horseshoe bats. This suggested that the primary host of SARS-CoV-2 was a bat and other animals including pigs, or pangolins are potential secondary hosts to the virus (Lai et al., 2020; Sun et al., 2020).

To date, the impact of the seafood market in spreading COVID-19 is not fully understood (Harapan et al., 2020). It has been proposed that SARS-CoV-2 was introduced to the seafood market in Wuhan, Hubei, China and then the disease spread more rapidly through human-to-human interactions, which has been confirmed by the occurrence of infection among the family members and medical workers attending the victims (Chan et al., 2020; Yu et al., 2020). The most plausible transmission routes are respiratory droplets dispersed via talking, sneezing and coughing or direct contact with infected persons. Other suggested routes are fecal-oral transmission, contaminated fomite transmission and mother-fetal vertical transmission (Naserghandi et al., 2020; Wang et al., 2020).

It has been demonstrated that COVID-19 can also be transmitted by asymptomatic patients who are harboring the virus during its early incubation period before symptoms appear (Li et al., 2020; Ye et al., 2020; Zhang et al., 2020). Additionally, SARS-CoV-2 has reappeared in recovered patients leading to a reoccurrence of illness. This has been confirmed through the detection of viral nucleic acid using a real-time RT-PCR after patient recovery and discharge from the hospital (Chen et al., 2020; Lan et al., 2020; Qiao et al., 2020). However, the exact reason for the reappearance of SARS-CoV-2 is not well understood.

## Discussion

### The Potential COVID-19 Transmission via Food Products

Foodservice operators were among the first workers in frontline employment sectors experiencing the impact of the COVID-19 pandemic. However, there is no study to date which reports that COVID-19 spreads via food products. Further, no evidence is available showing that viruses which infect the respiratory tract can be transmitted via food or food packaging (Food and Agriculture Organization of the United Nations [FAO] and World Health Organization [WHO], 2020). The transmission of SARS-CoV and MERS-CoV through the consumption of foods does not appear to have occurred yet (European Food Safety Authority [EFSA], 2020). However, it has been reported that human coronavirus 229E (HuCoV-229E) survived for at least 5 days on the surfaces of polyvinyl chloride (PVC), polyfluorotetraethylene (Teflon, PTFE), glass, ceramic tiles, and stainless steel and for 3 days on silicon rubber surfaces at 21°C with a relative humidity of 30–40% (Warnes et al., 2015). Similarly, SARS-CoV-2 survived on stainless steel and plastic up to 2 and 3 days, respectively, at 21–23°C and a relative humidity of 40%; however, the virus was not detected on copper and cardboard, after 4 and 24 h, respectively (van Doremalen et al., 2020). These results indicated that SARS-CoV-2 can be transmitted via contact surfaces because of the ability of the virus to survive on the surfaces for several days.

Coronaviruses can persist for long periods in environmental samples which may enhance the probability of transmission via package contact surfaces (Geller et al., 2012). It has been confirmed that virulence of variola (smallpox) virus and influenza virus were positively correlated with survival time in the external environment, which explained their high mortality rate compared to other viruses with low survival rates in environmental samples such as the virus causing parainfluenza and rhinovirus (Walther and Ewald, 2004).

Food and Agriculture Organization of the United Nations [FAO] and World Health Organization [WHO] (2020) proposed that touching food packages or containers contaminated with SARS-CoV-2 could transmit the virus to the mouth, nose, or eyes. However, this is not considered the main route for disease spread because the virus shows poor survival on these surfaces. A previous study reported that food products were a plausible transmission route for respiratory viruses including SARS-CoV-1 and influenza (Klein, 2004). In another study, the risk of Ebola infection to individual humans in the United States resulting from contaminated cocoa beans, palm oil, or cashews imported from South Africa was considered negligible to low (Bergeron et al., 2016). In addition, several studies showed that transmission of avian influenza through poultry products (Golden et al., 2009; Bauer et al., 2010; Sánchez-Vizcaíno et al., 2010) or water consumption was a remote possibility, but possible (Schijven et al., 2005). Similarly, the probability that consumers from the United Kingdom might get infected with COVID-19 via the consumption of food or the handling of material contacting food or packaging was considered negligible to very low. As mentioned, the genome of SARS-CoV-2 is closely related to SARS-CoV for which the transmission via foods has not been confirmed. It has been suggested that the potential foodborne transmission of SARS-CoV-2 may occur due to the consumption of foods originating from infected animals or the consumption of cross-contaminated foods (Oakenfull and Wilson, 2020).

More effort is needed to address the transmission of SARS-CoV-2 from the respiratory tract to food package surfaces or through food consumption. The Food and Drug Administration [FDA] (2020b) proposed guidelines for consumers during food shopping, food handling and food preparation. It is worth mentioning that food handlers including food establishment employees and consumers should adhere to good sanitation and hygienic practice guidelines to avoid SARS-CoV-2 transmission and comply with at least the minimum requirements of the food safety system.

### The Potential Survival of SARS-COV-2 in Food Products

It is widely known that viruses cannot multiply in food products because they need an animal or human host to grow. However, to date, no study has investigated the survival of SARS-CoV-2 in foods. To the best of our knowledge, only two studies reported the survival of infectious respiratory viruses in food products. Adenovirus survived on both lettuce and strawberries at 4°C for up to 10 days. In contrast, coronavirus survived only 2 days on lettuce, and it was not recovered from the surface of strawberries after inoculation (Yépiz-Gómez et al., 2013). These results indicated that respiratory viruses may transfer from food surfaces to the hands and subsequently to the mouth, nose or eyes. The survival of MERS-CoV in different types of milk (camel, goat and cow milk) at 4 or 22°C has been investigated. MERS-CoV titers were decreased by less than 1 log in all types of milk after 72 h at 4°C. Higher log reductions were observed when milk was stored at 22°C since the virus titers decreased by ≤ 2.0 log with 48 h of storage. Low temperature, long time pasteurization (63°C/30 min) of raw milk completely eliminated the virus from the milk of the three different animals (van Doremalen et al., 2014).

The infectious dose of most respiratory viruses is low; thus, the handling or consumption of food products could represent a risk for infection. As a result, preventive measures such as washing and sanitizing of fresh produce surfaces as well as the implementation of good personnel hygiene and practices among workers would seem reasonable ways to reduce the risk of virus transmission.

Another issue is that several viruses that cause respiratory infections have been found in the human gastrointestinal tract and were capable of proliferation there. These include *Enterovirus* (Coxsackie A, B virus), *Parechovirus*, *Orthomyxovirus* (Avian influenza virus), *Henipavirus* (Nipahand Hendra viruses), *Mastadenovirus* (adenovirus), *Alphatorquevirus* (Torque Teno virus), and coronavirus (Bosch et al., 2018). In the later case, lymphocytes and mucosal epithelial cells of the patients’ intestine were positive for SARS-CoV (Shi et al., 2005). This may mean that the viruses can be acquired by humans via the consumption of contaminated foods. However, these results may not apply to SARS-CoV-2, which points out the need for studies to investigate the survival of SARS-CoV-2 in different foods and on food packages.

### Need for Food Hygiene Practices From Farm to Fork

As mentioned, COVID-19 virus has an ability to stay alive for up to 72 h as a virion on inanimate objects after completing its life cycle in the body of an infected person (van Doremalen et al., 2020). Therefore, if the respiratory discharges of the COVID-19 patient come in contact with food, the food items can become a fomite (carrier), and if these items are contacted by other individuals, the virus is more likely to gain entry to their respiratory epithelium when unsanitized hands touch the nose, eyes, and mouth (Bundesinstitut für Risikobewertung [BfR], 2020; Centers for Disease Control and Prevention [CDC], 2020).

The surfaces of utensils, packaging material, counters, conveyor belts, interiors of transport vehicles, and all other food work stations where there might be human contact with food should remain a focus of attention where food handlers can act to impede the spread of COVID-19. Therefore, the proper use of personal protective equipment and adherence to the guidelines issued by public health authorities that include regular hand washing when exchanging goods, plus the use of hand sanitizers, wearing masks and gloves, and the maintenance of at least 6 feet between personnel are most important. A range of disinfectants and sanitizers are available in the marketplace. If disinfectant labels suggest that they are effective against coronaviruses or norovirus, then they should be effective against SARS-CoV-2 as well. Additionally, complete instructions are given on EPA disinfectant labels regarding the contact time, concentration, and appropriate surfaces for application (Environmental Protection Agency, 2020).

### Food Industries

All food industry organizations should strictly follow the protocols of Food Safety Management Systems (FSMS) given by authorities based on HACCP principles and should be kept updated in response to new pieces of evidence for viruses when required. In food companies where HACCP protocols are not being implemented, an expert should be appointed who will remain in contact with public health authorities to seek advice during the pandemic situation. Hand washing stations should be maintained for the workforce with the provision of normal soap, warm running water, hand sanitizers, and posters designed for displaying information regarding effective hand washing and sanitization. The physical distancing of 6 feet should be implemented among workers as infected people may remain asymptomatic or be pre-symptomatic during the course of the disease and may spread the infection when close to others (Kimball et al., 2020; Pan et al., 2020; Tong et al., 2020; Wei et al., 2020; Yu et al., 2020). The introduction of staggered workstations is an effective method to overcome the challenge of physical distancing in food industry facilities.

### Food Delivery

It is advised to minimize the contact between people during the outbreak; therefore, online food deliveries are more desirable. These allow physical distancing between customers and sales personnel. At this stage, proper dissemination of information on food handling practices is also required. Since food packages and paper currency are exchanged between consumers and retailers, proper precautions are needed to minimize the potential for virus transfer during the transaction. Some third-party delivery companies have also introduced contact-free delivery to homes. The packaging can be discarded after keeping track of important information mentioned on it. The proper use of gloves, sanitizers, and disinfectants can minimize the risk of virus spread and disease transmission (Food and Agriculture Organization of the United Nations [FAO] and World Health Organization [WHO], 2020; Food and Drug Administration [FDA], 2020a).

### Retail Food Premises

Maintaining the movement of food along the food chain is an important function that requires all involved to contribute and stay vigilant. It is necessary to maintain the confidence and trust of consumers regarding food safety and food availability. In case of limitations on the foodservice industry, home deliveries can be promoted, however, a safe and secure environment for food retail shops and canteens must be ensured at both the consumer’s and retailer’s end. Retailers can play their roles by ensuring the provision of sanitary facilities including wipes, disinfectants, sanitizers, and display of sanitary practices through visual aids. Physical distancing can be maintained by having the floors marked as a reference for maintaining minimum distance required. Plexiglass can be installed to avoid contact at cashier counters, and food tasting for promotional campaigns should be avoided (Food and Agriculture Organization of the United Nations [FAO] and World Health Organization [WHO], 2020; Food and Drug Administration [FDA], 2020a).

At the consumer end, people should make sure that family members belonging to vulnerable groups (immune-compromised, elderly, children, and COVID-19 patients) should stay at home (Centers for Disease Control and Prevention [CDC], 2020). Besides, the use of masks, wearing gloves, using hand sanitizers, using wipes before handling food carts, avoiding reusable shopping bags and opting for acceptable respiratory etiquette should be prioritized. If reusable bags are deemed regionally acceptable, their disinfection should be done instantly after use.

### Home Kitchens

It is advised to stock food items according to their perishability in order to minimize the number of visits to markets. After safe purchasing with all protocols, the most neglected thing is the safe handling of food. With respect to food handling practices in our kitchens, evidence that has been obtained with coronaviruses indicated that they cannot survive cooking, but if food remains unwashed and is frozen afterward, the virus can survive up to 2 years during frozen storage (Bundesinstitut für Risikobewertung [BfR], 2020). Therefore, scrubbing of food items like fruits and vegetables is essential if they are not to be cooked for longer periods or to be eaten uncooked. For canned foods, the lids should be wiped before opening. Ensure the disinfection of utensils, pots, countertops, and fridge at every use and minimize the risks of cross-contamination among food items during storage. In addition, respect cooking protocols to ascertain food safety and avoid a false sense of security by following proper cooking time, temperature, and thawing protocols.

### Need for Active and Intelligent Packaging

Consumers’ concern regarding the ability of SARS-CoV-2 to survive on the surface of packages has led to an increasing interest in the development of polymers and biopolymers with antiviral properties. The applications of polymers and biopolymers have shown high efficacy against hepatitis A virus (HAV) and human norovirus (HuNoV) (Randazzo et al., 2018). A previous study showed that the release of copper ions can help in the inactivation of HuCoV-229E on copper or copper alloy surfaces (Warnes et al., 2015). These findings are a source of validation for the recent findings by van Doremalen et al. (2020) regarding the decreased viability of SARS-CoV-2 on copper surfaces and inactivation within 2 h. The lack of trials on food matrices and related food regulatory requirements remain the hurdles to the adoption of novel food packaging. The development of biopolymers with antiviral properties and their applications in the food area remains an open field of research. For example, it has been recently reported that the use of nanomaterial coatings or films containing copper, silver, and zinc nanoparticles has a potential against SARS-CoV-2 to prevent contamination of food packaging surfaces and thus reduce its transmission (Sportelli et al., 2020).

In conclusion, there are only shreds of evidence regarding the duration of coronavirus survival on different contact surfaces and in foods under certain conditions which suggests the need for advanced studies in understanding the risk of COVID-19 spread associated with food and food packages. Research trials are required to find a link between the ingestion of food contaminated with SARS-CoV-2 and the probability of infections as well as the development of antiviral active packaging using nano-based biopolymer materials. The current guidelines issued by public health authorities are based on the disease patterns of previously encountered coronaviruses and they need to be updated according to the novel coronavirus SARS-CoV-2 as this virus is likely to persist and people will have to modify their “normal behavior” to a “new normal.”

## Data Availability Statement

All datasets generated for this study are included in the article/supplementary material.

## Author Contributions

AO, HS, and NF wrote the draft of the manuscript. AO, HS, NF, and SM reviewed and edited the final manuscript version. RH critically revised the final manuscript version. All authors contributed to the article and approved the submitted version.

## Conflict of Interest

The authors declare that the research was conducted in the absence of any commercial or financial relationships that could be construed as a potential conflict of interest.
